# AI-based approaches in the daily practice of abdominal imaging

**DOI:** 10.1007/s00330-023-10116-1

**Published:** 2023-08-09

**Authors:** Sabine Schmidt

**Affiliations:** Department of Diagnostic and Interventional Radiology, Lausanne University Hospital (CHUV) and University of Lausanne (UNIL), Rue du Bugnon 46, 1011 Lausanne, Switzerland

Over the last 7 years, the list of healthcare companies offering AI solutions and the number of artificial intelligence (AI) tools for radiology has expanded dramatically [1]. Claiming that AI applications can not only support radiologists in their daily work but will also even replace them in the end, some health-care professionals initially prophesied an “AI revolution” implying the imminent end of the traditional radiologist’s career as such. Indeed, compared with other hospital departments, radiology has traditionally held a leading position in introducing digital processes. However, the clinical implementation of AI in radiological departments has been moving slowly and seems disappointing to many of us. Despite an overwhelming offer proposed by numerous vendors, only few AI-based solutions have so far been integrated into diagnostic radiology, and even fewer in the daily practice of abdominal imaging. Two recent metanalyses [1, 2] reveal that abdominal imaging is the field where AI-based solutions have been the least frequently introduced compared with other organ-based specialities accounting for only 4% of commercially available AI-based applications, of which 3% dedicated to liver and 1% to prostate imaging, thus by far inferior to neuroradiology (29–38%), chest (24–31%), breast (11%), cardiac (11%), and musculoskeletal imaging (7–11%) [2]. Currently, AI-based tasks in abdominal imaging mostly focus on automated organ volumetry including segmentation or the detection and quantification of systemic diseases, such as liver fibrosis, hemochromatosis, or fatty liver [2]. In emergency imaging, only two scientific publications exist so far to the best of my knowledge. The first report on an AI algorithm previously trained to detect abdominal free gas, free fluid, and mesenteric fat stranding achieved an overall sensitivity of 93% and specificity of 97% [3], and the second on a deep learning–based system to detect renal stone disease on low-dose CT had a sensitivity of 86% [4].

Why do we observe such a delayed adoption of AI in our daily radiological practice, and why is there such a gap between abdominal imaging and the other anatomical regions? Firstly, AI applications are primarily narrow in terms of tasks, modality, and anatomic region. In contrast, abdominal radiologists need to be able to give diverse diagnostic answers simultaneously. This applies especially to emergency CT imaging where the on-call radiologist is required to exclude or confirm a myriad of possible abdominal diseases, often only with scarce clinical information. Furthermore, the emergency patient population is heterogeneous and imaging protocols vary considerably. Secondly, most abdominal radiologists perform their daily work with a picture archiving and communication system (PACS) in which the integration of several dozen AI-based solutions from different vendors running simultaneously is not only difficult but also costly. However, since each AI application needs to be thoroughly developed, tailored to the application and target environment, and thoroughly tested before it can be used in a routine clinical setting [5], this financial burden seems to be warranted. Thirdly, in general, AI tools represent highly technical and sophisticated products, not fully understood by each radiologist, and are, thus, mostly considered “black boxes.” This lack of transparency not only obscures the way AI tools reach a final diagnosis but also raises the question of the underlying medico-legal responsibility when any wrong conclusion resulting from their application occurs. This seems even more arguable since the public, and hence patients, do not (yet) seem to trust medical AI-based solutions. Fourthly, most AI tools are still work-in-progress; i.e., they are steadily learning and evolving thanks to continuous technological advancement. This may lead to a change in reproducibility over time. Finally, according to an online overview of commercially available (CE-marked) AI software products for clinical radiology based on vendor-supplied product specifications, only 36% of products have peer-reviewed evidence of which most studies demonstrate lower levels of efficacy [1].

Nevertheless, without the radiologists being necessarily aware of it, many non-interpretive AI solutions, although not limited to the abdomen, are already firmly established in the daily life of abdominal imaging. Running in the background of the radiology workflow, most of them improve diagnostic image quality. Furthermore, logistical AI tools, such as the selection of adequate imaging protocols before or the technical image quality control after data acquisition, have been developed [6, 7]. Deep-learning reconstruction (DLR) algorithms produce CT images with significantly less noise than when using iterative reconstructions, leading, thus, to increased contrast-to-noise ratio and improved lesion detectability [8]. In contrast-enhanced CT, DLR methods have been shown to significantly improve image spatial resolution and diagnostic confidence for the detection of hepatic lesions [9], and this even with the use of thin slices of 1.25 mm [10] while maintaining low radiation exposure [7]. Finally, streak artifacts produced from low-dose acquisition are also subtracted by means of DLR [7].

In conclusion, AI in abdominal imaging is arguably still even more in its infancy than observed in the other fields of clinical radiology. While non-interpretive AI tasks are being progressively deployed and implemented for any anatomical region, all-embracing diagnostic AI-based tools still need to be developed for clinical abdominal radiology, where many abdominal organs and various types of acute disease are considered simultaneously.

Today, abdominal radiologists face a tremendous increase in their daily workload, since nearly all patients presenting with acute abdominal pain undergo CT examinations. Therefore, they definitively need help from advanced AI tools assisting them with the final diagnosis. However, their development will be a long process, and much effort remains to be done to train these AI tools adequately. This training can and should only be done by radiologists together with AI experts. AI must give less narrow diagnostic answers and be able to flag any acute and/or suspicious condition; otherwise, AI will only complicate the daily life of radiologists. Starting with large datasets to each of the abdominal organs first considered separately and then merged to all-embracing AI-based tools, they may perform well even in the clinical field of abdominal radiology. However, the prerequisite of such a seamless integration in the daily workflow is that AI developers, health-care professionals, and patients trust each other and that the AI-based tools will be used to support radiologists and not instead of them.

## Declarations

### Guarantor

The scientific guarantor of this publication is Sabine Schmidt.

### Statistics and biometry

No complex statistical methods were necessary for this paper.

### Methodology

• commentary
